# Time and frequency domain methods for quantifying common modulation of motor unit firing patterns

**DOI:** 10.1186/1743-0003-1-2

**Published:** 2004-10-14

**Authors:** Lance J Myers, Zeynep Erim, Madeleine M Lowery

**Affiliations:** 1 Rehabilitation Institute of Chicago, Sensory Motor Performance Program, 345 East Superior St, Chicago, Illinois, 60611, USA; 2 Department of Physical Medicine and Rehabilitation, Feinberg School of Medicine, Northwestern University, Chicago, Illinois, USA

**Keywords:** coherence, common drive, motor unit discharge, descending drive

## Abstract

**Background:**

In investigations of the human motor system, two approaches are generally employed toward the identification of common modulating drives from motor unit recordings. One is a frequency domain method and uses the coherence function to determine the degree of linear correlation between each frequency component of the signals. The other is a time domain method that has been developed to determine the strength of low frequency common modulations between motor unit spike trains, often referred to in the literature as 'common drive'.

**Methods:**

The relationships between these methods are systematically explored using both mathematical and experimental procedures. A mathematical derivation is presented that shows the theoretical relationship between both time and frequency domain techniques. Multiple recordings from concurrent activities of pairs of motor units are studied and linear regressions are performed between time and frequency domain estimates (for different time domain window sizes) to assess their equivalence.

**Results:**

Analytically, it may be demonstrated that under the theoretical condition of a narrowband point frequency, the two relations are equivalent. However practical situations deviate from this ideal condition. The correlation between the two techniques varies with time domain moving average window length and for window lengths of 200 ms, 400 ms and 800 ms, the *r*^2 ^regression statistics (*p *< 0.05) are 0.56, 0.81 and 0.80 respectively.

**Conclusions:**

Although theoretically equivalent and experimentally well correlated there are a number of minor discrepancies between the two techniques that are explored. The time domain technique is preferred for short data segments and is better able to quantify the strength of a broad band drive into a single index. The frequency domain measures are more encompassing, providing a complete description of all oscillatory inputs and are better suited to quantifying narrow ranges of descending input into a single index. In general the physiological question at hand should dictate which technique is best suited.

## Introduction

Common oscillations in neurophysiological activity in the human motor system have been well documented. During voluntary muscle contraction, the human central nervous system drives motor neurons at a range of frequencies which cause common modulations in the firings of these neurons. These drives are reviewed in [1] and [2] where they are summarized into four broad frequency ranges: (1) A low frequency drive at around 1–3 Hz (2) A neurogenic component of physiological tremor that occurs between 5–12 Hz and is likely to have both spinal and supraspinal components. (3) A corticospinal drive in the beta (15–30 Hz) range (4) A corticospinal drive in the low gamma (30–60 Hz) range, that increases in importance with stronger contractions and is called the Piper rhythm.

There are two distinct approaches toward the identification of these drives. The majority of the literature has examined common modulation to motor units using frequency domain methods. This methodology was first introduced by Rosenberg and colleagues [3] and applied by Farmer and colleagues [4] who used coherence analysis to identify both a significant low frequency and beta-band association between motor unit firings in the 1–12 Hz and 15–30 Hz frequency ranges respectively. Subsequently, coherence analysis has become an established technique to study bivariate motor system measurements and a number of works have used this to investigate corticomuscular interactions [5-8,1]; tremor [9]; aging [10]; oscillatory drive in Parkinson's disease [11,12]; dystonia [13]; stroke [14]; and cortical myoclonus [15].

A separate body of literature has focused specifically on the low frequency common drive. This drive was first identified by De Luca and colleagues [16] who demonstrated that the firing rates of concurrently active motor units (MUs) were modulated in a highly interdependent manner. They low-pass filtered the impulse trains corresponding to MU firing times to obtain the time-varying mean firing rates which they high-pass filtered at 0.75 Hz. They then performed a time domain cross-correlation analysis between pairs of zero-mean signals representing the fluctuations in mean firing rates. Peaks occurring near the zero lag location in the normalized cross correlations implied that those firings rates were essentially simultaneously modulated with virtually no time delay. This phenomenon was termed 'common drive' to indicate a common excitation to the motor neuron pool that results in concurrent fluctuations in the firing rates of motor units from the same pool. A number of subsequent studies have utilized this technique to investigate the relationship of this drive to handedness [17-19]; different proprioceptive conditions [20]; exercise [21]; task and disease [22]; and aging [23]. These works have established this time domain method as an accepted means of quantifying the common low frequency modulation of MU firings.

In a recent review [1], it was suggested that the low frequency common drive first identified by De Luca and colleagues [16] using time domain methods is essentially the same low frequency drive as detected by Farmer and colleagues [4] using frequency domain methods. In this paper we explore the relationship between the two techniques using mathematical and experimental approaches.

## Analytic methods

### Frequency domain methods: Coherence

The coherence between two zero-mean stationary random processes *x*_1 _(*t*) and *x*_2 _(*t*), at frequency *f*, is defined as:



where  (*f*) is the cross spectral density and  (*f*) and  (*f*) are the auto spectral density functions of *x*_1 _(*t*) and *x*_2 _(*t*) respectively. The coherence function is a complex quantity and its squared magnitude provides a bounded measure of linear association between the two series, taking on a value of 1 for a perfect linear relationship and a value of 0 to indicate that the series are uncorrelated. In practice, we are often limited to a single time-limited realization of each random process and hence it is necessary to estimate the magnitude squared coherence, , by windowing the time series to obtain multiple sections as follows:



where * denotes complex conjugation, *N *is the number of data segments employed and X_1*n *_(*f*) and *X*_2*n *_(*f*) are the discrete Fourier transforms of the *n*th data segments of *x*_1 _(*t*) and *x*_2 _(*t*). This estimate is biased and its probability density function for non-weighted and non-overlapping windows has been analytically determined [24]. This may be used to derive the value of the estimated coherence, with a particular probability of occurrence, *α*, that would be obtained when the true value is zero. Any value exceeding this level is considered to be unlikely to be a false indication of coherence with (*α *× 100) % confidence. This confidence level is given by [24,3]

*E*_*α *_= 1 - (1 - *α*)^1/(*N*-1) ^    (3)

**Figure 1:**
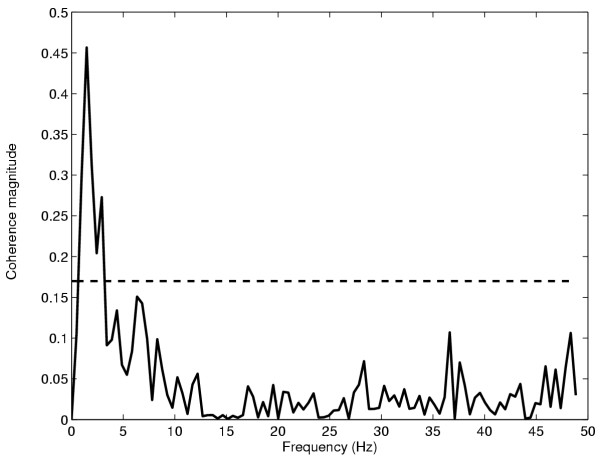
Example of a magnitude squared coherence plot. Magnitude coherence between two motor unit spike trains recorded from the FDI muscle. The dashed horizontal line indicates significance at the 95% confidence level. Significant coherence occurs between 0.5 and 3.5 Hz with the peak coherence of 0.46 occurring at 1.5 Hz.

The resolution of the coherence estimate is determined from the inverse of the length of the windowed sections, i.e., for a 2 s window, the coherence resolution will be 0.5 Hz. Figure 1 depicts a typical coherence plot computed for the spike trains of two MU's and the associated 95% confidence level. The coherence plots reveal the bandwidth and values of significant coherence for the given resolution. Results from coherence analyses are usually quantified in terms of either the peak value and its frequency or the frequency range of significant coherence. In Figure 1 there is significant coherence between 0.5 and 3.5 Hz and the peak value of coherence is 0.46 and occurs at 1.5 Hz.

### Time domain methods: Cross correlation

**Figure 2:**
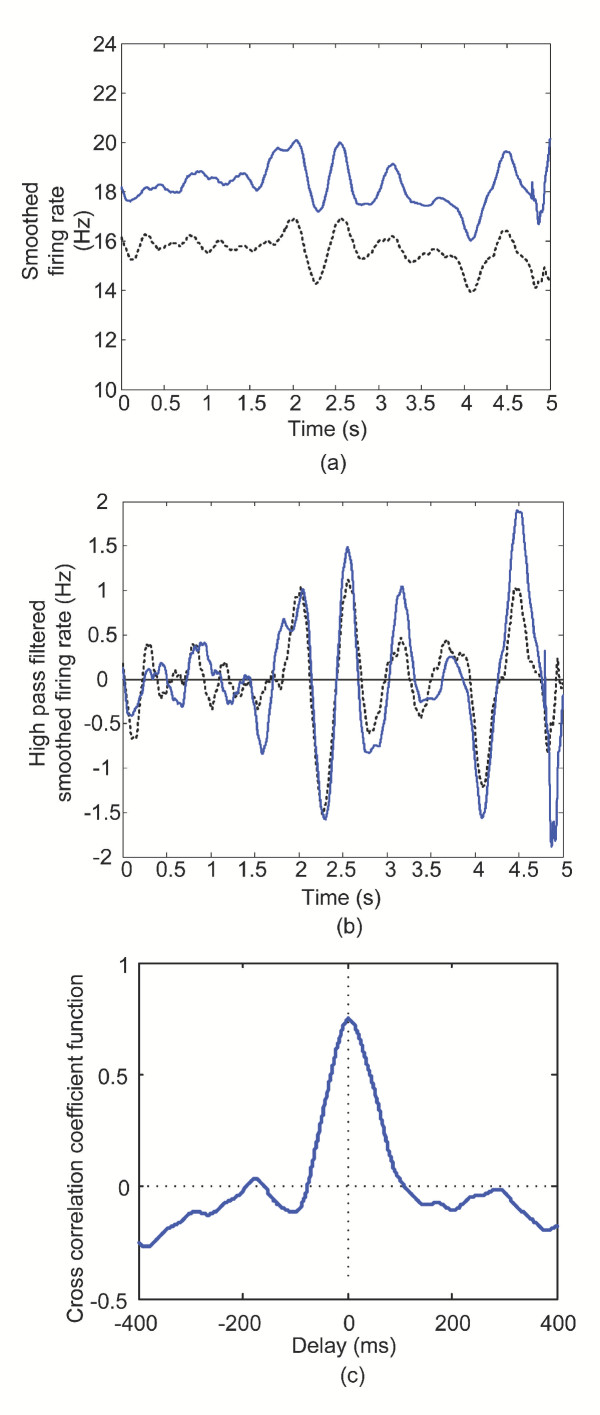
Example of the construction of a low frequency common drive plot. A low-pass, moving average Hanning window filter of length 400 ms was applied to two motor unit spike trains recorded from the FDI muscle. (a) A 5 s epoch of the time-varying smoothed firing rates; (b) the high-pass filtered version of the smoothed firing rates shown in (a); and (c) the low frequency common drive coefficient function between two motor unit spike trains. This results in an effective pass band of 0–5 Hz. The peak of the signal is 0.75 and occurs at a lag of 3.5 ms.

The cross correlation between two zero-mean stationary random processes *x*_1 _(*t*) and *x*_2 _(*t*) is defined as:



where *E *[·] is the estimation operator. Assuming ergodicity, for single time-limited realizations of each random process, this is determined using the integral:



where * denotes complex conjugation and *τ *is the time lag between the signals. The Fourier transform of the cross correlation function, defines the cross-spectrum,  (*f*). Cross correlation functions are unbounded measures and are typically normalized by the values of the autocorrelations at zero lag to bound the estimate between -1 and 1. The autocorrelation functions are the time domain equivalent of the auto power spectra and their value at zero lag represents the total energy in the signal. The normalized and bounded measure is known as the cross correlation coefficient, , which provides a measure of the linear association between the two signals at a given time lag and is given by:



The original method employed by De Luca and colleagues [16] represents the time series as a binary pulse train with ones corresponding to the firing times of the MUs and zeros comprising the remainder of the signal. A moving average window is then used to smooth these binary signals, which is analogous to filtering the time-series with a low-pass filter. These smoothed signals are depicted in figure 2a. A high-pass filter is then used to remove the mean bias of the signal as 'shown in figure 2b. The filtered signals are subsequently cross correlated and an index obtained from the peak value of the normalized cross correlation function within a specified lag window. Here we term this index the time domain common drive coefficient.

Figure 2c depicts the normalized cross correlation function for the same two motor unit spike trains used in Figure 1 obtained using a moving average Hanning window of 400 ms duration and high pass filtering at 0.75 Hz. The function is displayed for lags up to ± 400 ms and the peak of the signal indicated at a lag of 3.5 ms. The time domain common drive coefficient is measured as 0.75.

### Relationship between cross correlation and coherence

The cross correlation coefficient is related to coherence using a similar analysis to Gardner [25] as follows:

We begin with the real and stationary signals *x*_1 _(*t*) and *x*_2 _(*t*), where *x*_2 _(*t*) = *αx*_1 _(*t *- *τ*_0_) + *n *(*t*) is a scaled and time-delayed version of *x*_1 _(*t*), with additive uncorrelated, zero mean noise, *n *(*t*). The cross-correlation function is given by



since the cross correlation with the noise is zero everywhere. The cross spectrum is given by



Assume that the signals *y*_1 _(*t*) and *y*_2 _(*t*) result from passing the signals *x*_1 _(*t*) and *x*_2 _(*t*) respectively through a tunable narrowband bandpass filter with transfer function denoted by *H *(*f*):



where  and Δ are the center frequency and bandwidth of the ideal bandpass filter. The cross-correlation function between filtered signals *y*_1 _(*t*) and *y*_2 _(*t*) is given by:



where  (*f*) is the cross power spectral density function of *y*_1 _(*t*) and *y*_2 _(*t*).

The cross power spectrum may be written in terms of its magnitude and phase, . Since for stationary, real signals, the autocorrelation is real and even and hence,  (*f*) is real, the phase of the cross spectrum is given by (equation 8):



Thus replacing  (*f*) with |*H *(*f*)|^2 ^ (*f*) in equation 10 we get



since  is real for real *x*_1 _(*t*) and *x*_2 _(*t*).

Similarly for the autocorrelation functions we get



and



Thus as Δ → 0 we obtain the expression for the normalized cross correlation function as:



The peak of the cross-correlation function occurs at the time delay, *τ *= *τ*_0_. Thus



Thus we see that the peak of the normalized cross-correlation function between two signals after ideally bandpass filtering to contain a single frequency, is identical to the *magnitude *of the coherence function of the original signals at the frequency of the filter. The phase of the coherence function is the same as the phase of the cross-spectrum and provides the time delay.

For a less ideal filter that spans several frequencies the relationship is less precise and may be derived as follows:

Let *W *(*f*) be the new filter transfer function and thus the normalized cross-correlation function is:



where *f*_1 _and *f*_2 _are the cut-off frequencies of the filter. Thus when multiple frequencies are present, this may be thought of as taking the weighted summation of the cross-correlation functions at each frequency present and normalizing this by the product of the weighted summations of the autocorrelations across all frequencies present. The more narrow band the filter used, the more similar the time domain correlation and frequency domain magnitude coherence measures. As the filter encompasses a greater range of frequencies, measures from the two methods will increasingly deviate.

The low frequency time domain method employed by De Luca and colleagues [16] utilized a moving Hanning window as a low pass filter. The cut-off frequency of the filter is dependent on the time constant of the filter which is typically 400 ms [16,21] but values up to 0.95 s have also been used [26]. However different window lengths will modify the relationship between this time domain measure and the coherence function.

The effect of varying window length may be illustrated by obtaining an expression for the filter transfer function. The equation for the Hanning window is given as:



where *τ *is the length of the window. The discrete Fourier transform of this is given as (Kay, 1988):



where



**Figure 3:**
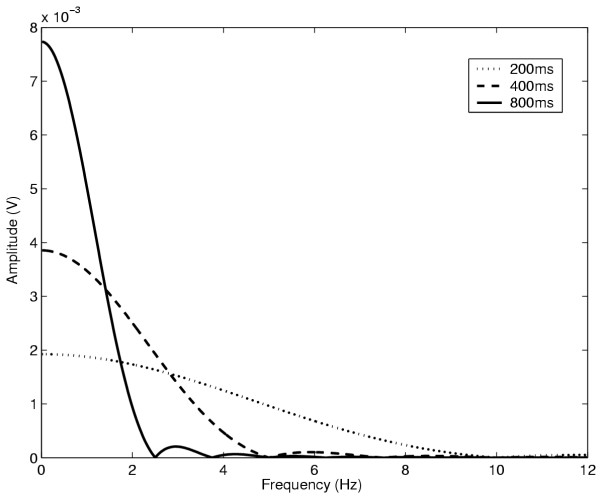
Magnitude squared spectra of Hanning window filters. Magnitude spectra of the transfer functions of Hanning window filters for three different time constants, *τ *= 200 ms (dotted line), *τ *= 400 ms (dashed line) and *τ *= 800 ms (solid line). As the time constant increases the bandwidth of the filter decreases and its magnitude increases.

Figure 3 depicts the transfer function power spectrum (|*W *(*f*)|^2^) for *τ *= 200, 400 and 800 ms. The figure clearly demonstrates that as the length of the analysis window decreases, the bandwidth of the filter increases. Therefore the only information that can be ascertained with shorter windows is that the frequency of the common modulating input lies somewhere within the frequency range specified by the window. Longer windows result in a better correlation with coherence values at lower single frequencies (close to zero), while shorter windows lump into a single value a weighted expression of the coherence values in the frequency range which they span.

## Experimental methods

In this section we demonstrate the relationship between time and frequency domain based methods to estimate the common modulating drive using empirical data. The methods are applied to data collected during isometric contractions of the First Dorsal Interosseous muscle at 20% of maximal effort. Two contractions where the activities of 4 and 5 MUs were identified yielded a total of 16 pairs of coactive MUs. The periods of concurrent activity of these MU pairs ranged between 30 s to 1 minute and were further divided into pairs of non-overlapping 10 s intervals resulting in a total of 50 pairs of 10 s long spike trains. Each method was applied to these spike train pairs and the correlation between the results yielded by the two methods were investigated as discussed below.

The time domain method was used to estimate low frequency common drive according to the method described by De Luca and colleagues [16]. Three different time domain estimates were formed by smoothing the spike trains using Hanning windows of length 200, 400 and 800 ms respectively. These smoothed firing rate signals were then digitally high pass filtered with a low frequency cut-off of 0.75 Hz using a third order Butterworth filter to remove the mean bias discharge rates. The cross correlation coefficient function of these high pass filtered records was then obtained and the peak value of this function within ± 50 ms of the zero time lag was recorded and termed the time domain common drive coefficient.

The coherence analyses were performed in a similar manner to the procedure of Rosenberg and colleagues [3] for point process data. The spike trains were represented as binary pulse trains with ones corresponding to the firing times of the MU's and zeros comprising the remainder of the signal. Fourier transforms of these trains were obtained for each appropriately windowed section and then averaged according to equation (2). However where Rosenberg and colleagues [3] do not use overlapping or tapered data windows, we used overlapping, tapered Hanning windows of 2048 ms to optimize the variance and bias of the estimate. With any non-parametric spectral estimation technique, there is a trade-off between the variance and both the bias and resolution of the estimation. A window size of 2048 ms, gives a frequency resolution of 0.49 Hz, which is adequate to discriminate frequencies for our purposes. However, when analyzing 10 s of data using 2048 ms non-overlapping windows, only 5 different records are available and this small number of records will increase the variance of the estimate. Furthermore rectangular windows introduce an estimation bias due to the effect of their sidelobes. These concerns may be reduced by using the Welch periodogram method which uses tapered windows (to reduce spectral leakage and therefore the estimation bias) and overlapping windows (to increase the total number of windows and hence reduce the variance). The minimum variance for this method is obtained using an overlap of 62.5% [27]. The frequency corresponding to the first zero-crossing of the Hanning filter was obtained according to equation (20) and the peak value of the coherence in the range between 0.75 Hz and this frequency was recorded.

A linear regression between the time domain common drive coefficients and corresponding frequency domain peak coherence values was performed to determine whether a linear relationship between the two indices existed. The regression *r*^2 ^values are reported at a significance level of *p *< 0.05.

## Results and discussion

**Figure 4:**
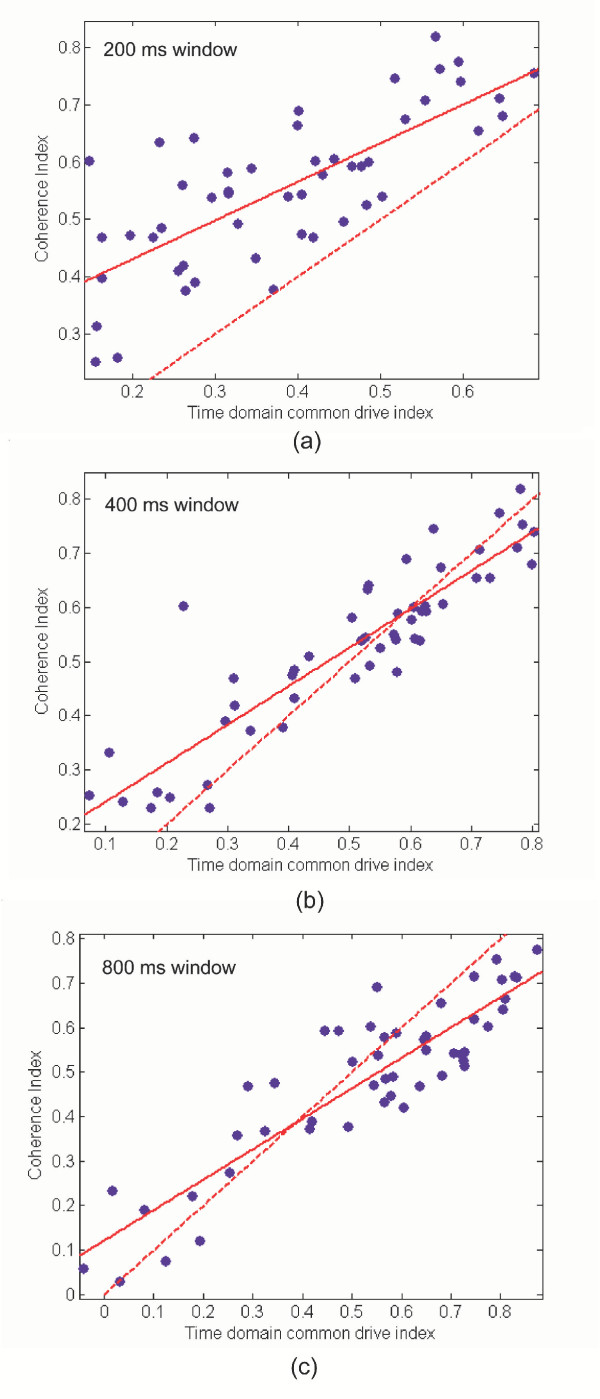
Regression plots for low frequency common drive time versus frequency domain techniques. Regression plots for low frequency common drive time versus frequency domain techniques. Three different moving average Hanning windows were used to low pass filter the time series for the time domain method. The time constants for the filters are as follows: (a) *τ *= 200 ms, (b) *τ *= 400 ms, (c) *τ *= 800 ms. All regressions are significant at *p *< 0.05 and the *r*^2 ^statistics are (a) 0.56, (b) 0.81 and (c) 0.80. The unitary slop line is indicated in the figures as a dashed line and represents the ideal mathematical relationship.

Figure 4a,4b,4c displays the regression between the low frequency time domain common drive coefficients for Hanning windows of length 200, 400 and 800 ms and peak low frequency coherence. All regressions are significant at *p *< 0.05 and the *r*^2 ^statistics are 0.56, 0.81 and 0.80 respectively. A unitary slope line is displayed in the figure and this describes the theoretical relationship between the two indices. These results indicate that for the larger 400 ms and 800 ms windows, the time domain method is more closely correlated with the coherence estimate, with the 400 ms window yielding a marginally better fit. The data for the smaller 200 ms window exhibits a consistent bias, with the coherence estimate larger than the time domain common drive estimate, whilst the 400 ms and 800 ms windowed data are more evenly distributed around the unitary slope line, indicating less bias. There are a number of possible factors that could contribute to the observed mismatches between the two methods.

As demonstrated in Figure 2, the cross-correlation peak can occur at lags slightly different than zero. A time delay or misalignment has been shown to introduce a bias into the coherence estimate that is proportional to the delay and coherence magnitude and inversely proportional to the FFT epoch duration [24]. However for delays of the order of magnitude of ± 50 ms and FFT lengths of approximately 2 s, this type of bias is very small and unlikely to account for the observed differences between the time and frequency domain estimates.

**Figure 5:**
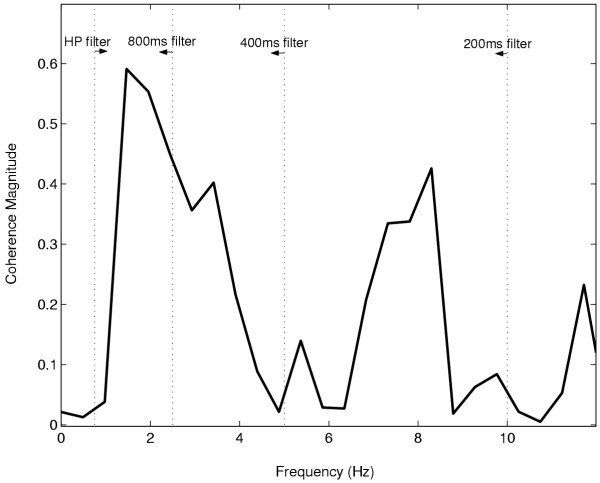
Magnitude squared coherence between two motor unit spike trains recorded from the FDI muscle. Magnitude coherence between two motor unit spike trains recorded from the FDI muscle. The vertical dotted lines from left to right represent the cut-off frequencies of the 0.75 high-pass filter, and the 800 ms, 400 ms and 200 ms moving average low-pass filters. The peak coherence occurs at 1.5 Hz.

The use of a short duration window in the time domain method results in the inclusion of multiple frequencies in the time domain correlation estimation according to equation (18). The bandwidths of descending oscillatory drives may be variable. Thus when the descending drive occupies a narrow bandwidth and the time domain window includes a greater range of frequencies than this bandwidth, this will bias the time domain estimate to be lower than the peak coherence value as is the case in figure 4a. Alternatively should the drive span a broader bandwidth, the time domain measure would encompass all the correlated frequencies into a single value and would thus be different than the value obtained from any single peak coherence frequency. This idea is illustrated in Figure 5 where a typical coherence plot is displayed. Superimposed on this are vertical lines representing various moving average filter cut-off frequencies. The 0.75 Hz high pass cut-off frequency is also displayed. Thus from the figure we see that in this case the coherence occupies a fairly broad bandwidth from 1–5 Hz, peaking at 1.5 Hz. The cut-off frequency of the 200 ms filter is approximately 10 Hz and thus the time domain estimate will include coherence values at all these frequencies which would make it significantly different from the peak coherence. The 400 ms and 800 ms windows would better correlate with the peak coherence frequencies and the 400 ms window would provide a better overall index encompassing the full bandwidth of the drive. However, if the middle peak at 8 Hz were stronger and actually the main peak, the wider time windows would miss it altogether. This emphasizes the importance of *a priori *knowledge in choosing the appropriate time windows in the time-domain based method. Therefore in summary, the time domain measure is more effective in quantifying a range of frequencies into a single index and the peak coherence estimate is better at representing the coherence at any single frequency.

Coherence estimates are typically formed from data records of around 1–5 minutes in length [4,28,29] or from pooled coherence measures of repeated trial measurements [30]. This increases the number of non-overlapping windows in the calculation, thereby reducing the variance of the coherence estimate. Non-overlapping, rectangular windows are traditionally preferred due to the clear relationship with significance levels. Overlapping, tapered windows will allow coherence to be estimated from shorter data segments and parametric techniques, in particular multivariate autoregressive (MAR) methods are suggested for the analysis of very short duration data segments [31]. When using short records of data (<5 s), the coherence estimates are likely to be significantly biased. However, the time domain method is more robust for such short data lengths and would therefore be preferred in these situations.

The time domain method uses a high pass filter to remove the mean bias from the smoothed signals, whereas the frequency domain coherence method simply subtracts the mean component of the signal prior to forming the estimate. Although similar, these two methods are not identical and may further explain some of the variation between the time and frequency domain techniques. A further possibility is to employ a low order polynomial detrending technique instead of high pass filtering or subtracting the mean. In general, a visual examination of the smoothed firing rate signals would indicate whether this would be necessary.

It is straight forward to quantify any time delay using the time domain technique. Although this is also possible with the frequency domain technique, this delay information is incorporated in the phase of the estimate and is therefore 2*π *periodic and would thus yield the same result for integer multiples of delay. For significant coherence present over a band of frequencies, Mima and colleagues [32] suggest a constant phase shift plus constant time delay regression model to compute time delays from coherence estimates. However for narrow band descending drives, the time domain technique provides a clearer estimate of any time delay.

It should be noted that the time domain technique may be generalized to cover any arbitrary frequency. This would necessitate bandpass filtering the signals to the frequency range of interest, removing the mean trends and then evaluating the cross-correlation function. Although this requires *a priori *knowledge of the drive bandwidth, this method would then be able to provide a single index to quantify a particular bandwidth.

## Conclusions

Two separate bodies of literature offer techniques to estimate band limited common oscillatory input to motor neurons. These techniques are based in either the time or in the frequency domain. Indices derived from both measurement techniques are well correlated with each other and in the theoretical limit, the techniques are shown to be mathematically equivalent. However, for practical purposes there are a number of minor discrepancies which may favour the use of one particular method for a given application.

The time domain method offers greater resolution in time (the latency of the correlations are easily revealed) at the expense of the requirement of *a priori *knowledge of the bandwidth of the common modulating drive. On the other hand the frequency domain technique reveals information regarding the frequency distribution of the common modulating drives but it is more difficult to obtain accurate estimates of the coherence with short signal lengths as well as of the exact delay.

Time domain methods of estimation are preferred for short data segments and are well suited to quantifying the strength of a broad band drive into a single index. This proves useful in quantitative, comparitive analyses of the common behavior of MUs such as statistical tests that investigate the effects of aging or disease. Frequency domain measures tend to be more encompassing as they provide a complete description of all common oscillatory inputs. This facilitates qualitative analysis of distribution of coherence across frequencies and hence leads to a better understanding of the nature of the common inputs. They are well suited for estimation from large data segments, that may be assumed to be stationary, and are better able to quantify narrow ranges of descending inputs into a single index. Thus the selection of one technique over another should be dictated by the nature of the physiological question to be addressed.

## References

[B1] Brown P (2000). Cortical drives to human muscle: the Piper and related rhythms. Prog Neurobiol.

[B2] Grosse P, Cassidy MJ, Brown P (2002). EEG-EMG, MEGEMG and EMG-EMG frequency analysis: physiological principles and clinical applications. Clin Neurophysiol.

[B3] Rosenberg JR, Amjad AM, Breeze P, Brillinger DR, Halliday DM (1989). The Fourier approach to the identification of functional coupling between neuronal spike trains. Prog Biophys Mol Biol.

[B4] Farmer SF, Bremner FD, Halliday DM, Rosenberg JR, Stephens JA (1993). The frequency content of common synaptic inputs to motoneurones studied during voluntary isometric contraction in man. J Physiol.

[B5] Conway BA, Halliday DM, Farmer SF, Shahani U, Maas P, Weir AI, Rosenberg JR (1995). Synchronization between motor cortex and spinal motoneuronal pool during the performance of a maintained motor task in man. J Physiol.

[B6] Salenius S, Portin K, Kajola M, Salmelin R, Hari R (1997). Cortical control of human motoneuron firing during isometric contraction. J Neurophysiol.

[B7] Halliday DM, Conway BA, Farmer SF, Rosenberg JR (1998). Using electroencephalography to study functional coupling between cortical activity and electromyograms during voluntary contractions in humans. Neurosci Lett.

[B8] Mima T, Hallett M (1999). Corticomuscular coherence: a review. J Clin Neurophysiol.

[B9] McAuley JH, Marsden CD (2000). Physiological and pathological tremors and rhythmic central motor control. Brain.

[B10] Semmler JG, Kornatz KW, Enoka RM (2003). Motor-unit coherence during isometric contractions is greater in a hand muscle of older adults. J Neurophysiol.

[B11] Brown P, Marsden J, Defebvre L, Cassim F, Mazzone P, Oliviero A, Altibrandi MG, Di Lazzaro V, Limousin-Dowsey P, Fraix V, Odin P, Pollak P (2001). Intermuscular coherence in Parkinson's disease: relationship to bradykinesia. Neuroreport.

[B12] Salenius S, Avikainen S, Kaakkola S, Hari R, Brown P (2002). Defective cortical drive to muscle in Parkinson's disease and its improvement with levodopa. Brain.

[B13] Farmer SF, Sheean GL, Mayston MJ, Rothwell JC, Marsden CD, Conway BA, Halliday DM, Rosenberg JR, Stephens JA (1998). Abnormal motor unit synchronization of antagonist muscles underlies pathological co-contraction in upper limb dystonia. Brain.

[B14] Mima T, Toma K, Koshy B, Hallett M (2001). Coherence between cortical and muscular activities after subcortical stroke. Stroke.

[B15] Grosse P, Guerrini R, Parmeggiani L, Bonanni P, Pogosyan A, Brown P (2003). Abnormal corticomuscular and intermuscular coupling in high-frequency rhythmic myoclonus. Brain.

[B16] De Luca CJ, LeFever RS, McCue MP, Xenakis AP (1982). Control scheme governing concurrently active human motor units during voluntary contractions. J Physiol.

[B17] Kamen G, Greenstein SS, De Luca CJ (1992). Lateral dominance and motor unit firing behavior. Brain Res.

[B18] Semmler JG, Nordstrom MA (1995). Influence of handedness on motor unit discharge properties and force tremor. Exp RBraines.

[B19] Adam A, De Luca CJ, Erim Z (1998). Hand dominance and motor unit firing behavior. J Neurophysiol.

[B20] Garland SJ, Miles TS (1997). Control of motor units in human ffexor digitorum profundus under different proprioceptive conditions. J Physiol.

[B21] Semmler JG, Nordstrom MA, Wallace CJ (1997). Relationship between motor unit short-term synchronization and common drive in human first dorsal interosseous muscle. Brain Res.

[B22] Patten C, Kamen G (2000). Adaptations in motor unit discharge activity with force control training in young and older human adults. Eur J Appl Physiol.

[B23] Erim Z, Beg MF, Burke DT, de Luca CJ (1999). Effects of aging on motor-unit control properties. J Neurophysiol.

[B24] Carter GC (1987). Coherence and time delay estimation. Proc IEEE.

[B25] Gardner WA (1992). A unifying view of coherence in signal processing. Signal Processing.

[B26] De Luca CJ, Erim Z (1994). Common drive of motor units in regulation of muscle force. Trends Neurosci.

[B27] Marple SL (1987). Digital spectral analysis with applications.

[B28] Kristeva-Feige R, Fritsch C, Timmer J, Lucking CH (2002). Effects of attention and precision of exerted force on beta range EEG-EMG synchronization during a maintained motor contraction task. Clin Neurophysiol.

[B29] Gross J, Tass PA, Salenius S, Hari R, Freund HJ, Schnitzler A (2000). Cortico-muscular synchronization during isometric muscle contraction in humans as revealed by magnetoencephalography. J Physiol.

[B30] Amjad AM, Halliday DM, Rosenberg JR, Conway BA (1997). An extended difference of coherence test for comparing and combining several independent coherence estimates: theory and application to the study of motor units and physiological tremor. J Neurosci Methods.

[B31] Cassidy MJ, Brown P (2002). Hidden Markov based autoregressive analysis of stationary and non-stationary electrophysiological signals for functional coupling studies. J Neurosci Methods.

[B32] Mima T, Hallett M (1999). Electroencephalographic analysis of cortico-muscular coherence: reference effect, volume conduction and generator mechanism. Clin Neurophysiol.

